# Effective Cataract Surgical Coverage in a Rural Area of North India

**DOI:** 10.7759/cureus.84684

**Published:** 2025-05-23

**Authors:** Sumit Malhotra, Praveen Vashist, Rama S Rath, Noopur Gupta, Kalaivani Mani

**Affiliations:** 1 Centre for Community Medicine, All India Institute of Medical Sciences, New Delhi, IND; 2 Department of Community Ophthalmology, All India Institute of Medical Sciences, New Delhi, IND; 3 Department of Community and Family Medicine, All India Institute of Medical Sciences, Gorakhpur, IND; 4 Department of Ophthalmology, All India Institute of Medical Sciences, New Delhi, IND; 5 Department of Biostatistics, All India Institute of Medical Sciences, New Delhi, IND

**Keywords:** cataract, effective surgical coverage, india, older adult, rural

## Abstract

Introduction: Worldwide, only 17% of individuals affected by blindness and visual impairment have received access to quality cataract surgery. The primary objective of this study was to determine the Cataract Surgical Coverage (CSC) and the effective Cataract Surgical Coverage (eCSC) among older adults aged 50 years and above.

Methods: This community-based cross-sectional study was conducted among older adults in two blocks of Jhajjar district, Haryana. A total of 34 villages were selected based on probability proportionate to size. During Phase 1, house-to-house screening and referral of individuals with visual acuity less than 6/12 were conducted. Phase 2 involved visual acuity assessment and lens examination in a makeshift clinic. CSC (persons) and eCSC (persons) were calculated using the standard formula for three visual impairment cut-offs: less than 6/18, less than 6/60, and less than 3/60.

Results: Of the 2025 older adults surveyed, 1544 were referred to the makeshift clinic, and complete lens status data were available for 1420 participants. The CSC (persons) rates were 72.2% (95% CI: 69.9 to 74.5), 83.1% (95% CI: 81.2 to 85.0), and 88.0% (95% CI: 86.3 to 89.7), and the eCSC (persons) rates were 43.9% (95% CI: 41.3 to 46.5), 52.3% (95% CI: 49.7 to 54.9), and 55.1% (95% CI: 52.5 to 57.7) for participants with presenting visual acuity less than 6/18, less than 6/60, and less than 3/60, respectively.

Conclusions: The proportions of CSC were found to be higher than those of eCSC in the study area. The lower eCSC suggests that the quality of cataract surgical services may be suboptimal. Further investigation into the quality of surgical services may be required to improve the eCSC.

## Introduction

Cataract is one of the most common causes of avoidable blindness worldwide, particularly among individuals aged 50 years or older [1]. According to the Global Burden of Disease study, there are around one billion people globally with moderate or severe distance visual impairment. Of these, approximately 94 million have visual impairment due to cataract [1]. The proportion of cataract cases as a cause of moderate to severe visual impairment increased globally by 19.2% from 2010 to 2019, while in South Asia, the increase was 1.7% over the same period [2]. The prevalence of visual impairment is higher in low- and middle-income countries than in high-income countries. According to the National Blindness and Visual Impairment Survey conducted in India from 2015 to 2019, cataract was the leading cause of blindness, severe visual impairment, and moderate visual impairment among persons aged 50 years and above [3]. To address the burden of cataract-related blindness, various interventions have been implemented under the National Programme for Control of Blindness and Visual Impairment (NPCBVI) [4]. In 2019, approximately 6.6 million cataract surgeries were performed in India [5].

Cataract Surgical Coverage (CSC) is designated as a key parameter for assessing cataract surgical services at the community level [6]. It assesses the coverage of cataract surgical services among those who need them. However, the indicator remains silent about the quality of the services provided. Recognizing the importance of service quality, effective Cataract Surgical Coverage (eCSC) has also been included as an essential indicator, along with the measurement of cataract surgical coverage, in tracking progress toward achieving universal healthcare [7,8]. Several studies have reported cataract surgical coverage; however, few studies worldwide have reported on eCSC. This manuscript reports cataract surgical coverage and effective cataract surgical coverage, as well as their association with sociodemographic variables in a rural area of northern India.

## Materials and methods

**Figure 1:**
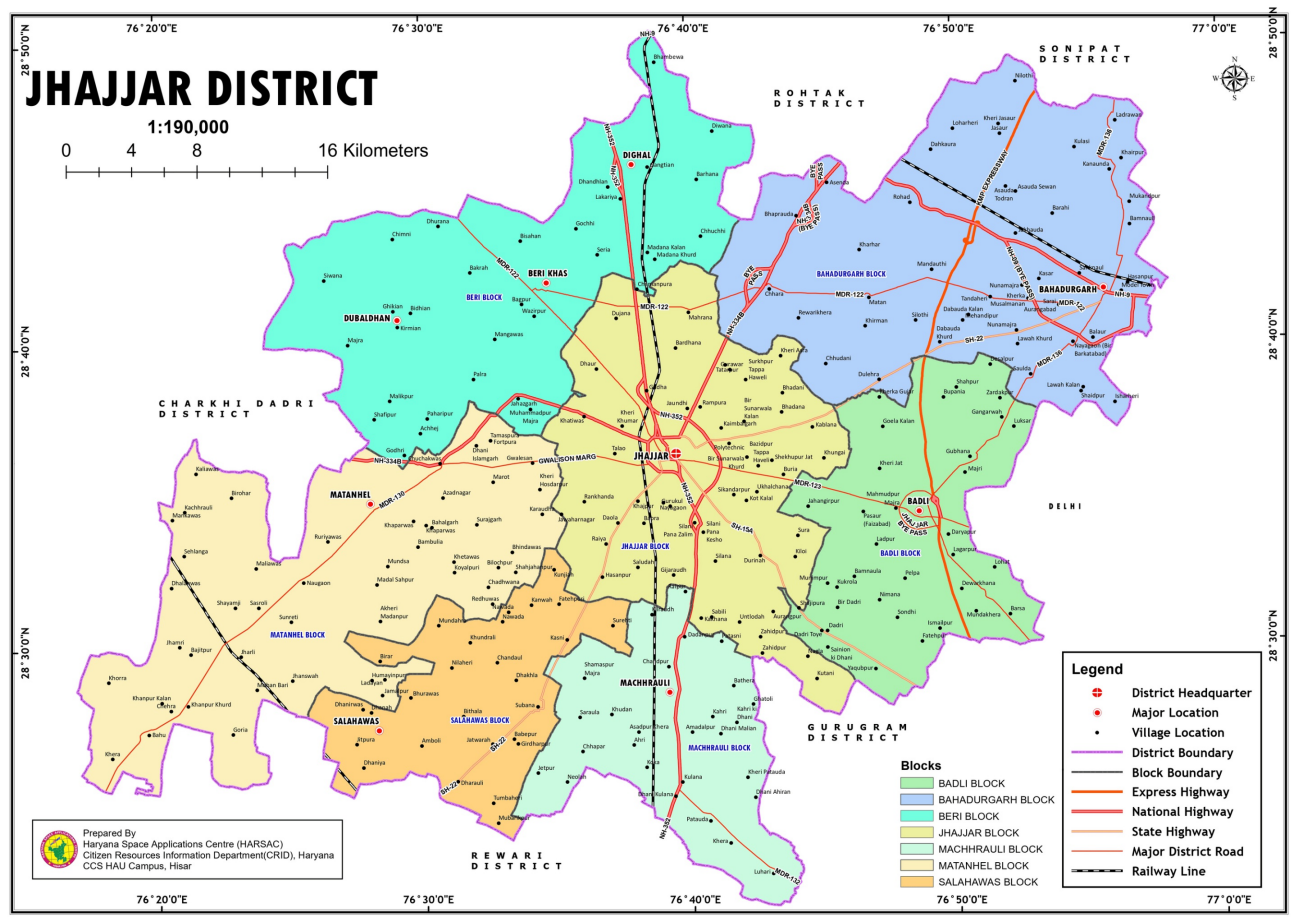
Study District, Jhajjar Map of the district has been obtained from district administration. Permission has been obtained from the district authorities to use the map.

The methods used in this study have been reported in detail elsewhere [9]. In brief, a community-based, cross-sectional study was conducted in two blocks of Jhajjar district, Haryana, India. The study district is shown in Figure 1. The district is 29 kilometers from Delhi, making it part of the National Capital Region. According to the 2011 census, the district had a population of 958,405, with the rural population constituting approximately 78%. The district implements blindness and visual impairment prevention and control strategies as mandated by the state government.

Two out of five blocks in the district (Bahadurgarh and Jhajjar) were included for the study. The rural areas were considered for inclusion due to their high prevalence of visual impairment. A list of all villages was prepared, and a total of 34 villages were selected using the probability-proportional-to-size sampling method. This study was a part of a more extensive study that was planned to determine the prevalence of visual impairment, and the computed sample size was 1,469 with the assumed prevalence of visual impairment in adults older than 50 years as 18.5%, relative precision of 15%, 25% non-response, and design effect of 1.5. Adults aged 50 years or older were selected using a compact segment cluster sampling approach. The survey team conducted the household visits, and the enumeration of adults was carried out within the chosen study segment of the study village. Written informed consent was obtained from the study participants. All the adults included in this study were considered for the objectives reported in this paper.

Presenting visual acuity (PVA) was measured at the household level by trained health assistants using a screening chart corresponding to five ‘E’ optotypes at 6/12. Correct identification of four out of five letters was considered the pass criterion. Presenting visual acuity was considered as vision with spectacles, if spectacles were used for distance vision. All participants with presenting visual acuity of less than 6/12 in either eye, as well as adults using spectacles and those with a history of previous cataract surgery, were referred to a temporary makeshift clinic within a village building, where optometrists performed a detailed eye assessment. The optometrist repeated the visual acuity measurement. Non-cycloplegic refraction was undertaken. The lens was assessed using a torchlight. A pupil that appeared gray or white when examined with oblique light was noted as a cataract. The absence of the lens (aphakia) and the presence of an intraocular lens (pseudophakia) were assessed and recorded.

The participants who did not visit the clinic after the referral had repeat visits made to their homes by the survey team on the same day in the afternoon to maximize their response, and they were encouraged to complete their examination process. The study epidemiologist and ophthalmologist rechecked 10% of all participants' forms and recorded vision findings as a quality assurance measure.

Operational definitions

There is some variation in the definition of cataract surgical coverage in published literature. We have used the following definitions [6,9].

CSC (persons) was defined as the number of people in a defined population with operated cataracts as a proportion of those with operable cataracts plus those who had undergone surgery. CSC (persons) was calculated using the following formula:

 

$$CSC = \frac{x+y}{x+y+z}\times 100$$

where *x* represents the individuals with unilateral pseudo/aphakia and operable cataract in the other eye; *y* represents the individuals with bilateral pseudo/aphakia, regardless of acuity; and *z* represents the individuals with bilateral operable cataract.

CSC (eyes) (visual acuity (VA)) was calculated using the formula:

 

$$CSC(eyes) = \frac{p}{p+q} \times 100$$

where *p* represents pseudo-aphakic eyes and *q* represents eyes with operable cataracts.

eCSC (persons) measures the number of people in a defined population with operated cataracts and a good outcome (i.e., presenting vision of 6/18 or better) as a proportion of those with operable or operated cataracts.

eCSC (persons) was calculated using the following formula: 

$$eCSC = \frac{a+b}{x+y+z}\times 100$$

where *a* stands for the individuals with unilateral pseudo/aphakia who achieve a presenting visual acuity of 6/18 or better in the operated eye and have an operable cataract in the other eye, and *b* represents the individuals with bilateral pseudo/aphakia who achieve a presenting visual acuity of 6/18 or better in at least one eye. *x*, *y*, and *z* are the same as for CSC.

Calculation of CSC and eCSC was performed for three visual acuity cutoffs: <3/60, <6/60, and <6/18.

All participants detected with vision impairment and a need for ophthalmic evaluation, especially those with poor outcomes in operated eyes, were referred for further management to the Ophthalmology Department at the All India Institute of Medical Sciences, Jhajjar complex. 

Data were entered using a Microsoft Access-based database with inbuilt consistency and validation checks. Statistical analysis was performed using Stata version 12.0 (StataCorp, College Station, Texas). Cataract surgical coverage and effective cataract surgical coverage were presented as proportions with 95% confidence intervals. CSC and eCSC were calculated for various subcategories of visual acuity. The relationship between various sociodemographic characteristics and CSC and eCSC was calculated using the chi-square test, with a p-value of 0.05 considered significant.

## Results

A total of 2,025 older adults were enumerated in the study area. Of these, 1,690 were examined at the household level. From these, 1,544 were referred to the clinic for examination. Of these, 1,429 reported to the examination site. The lens status of both eyes was available for 1,420 participants, and these data were used to calculate the cataract surgical coverage and effective cataract surgical coverage, as shown in Tables 1-3.

The cataract surgical coverage was found to be 72.2% (95% CI: 69.9, 74.5) among those whose PVA was less than 6/18, 83.1% (95% CI: 81.2, 85.0) among those with PVA less than 6/60, and 88.0% (95% CI: 86.3, 89.7) for those with PVA less than 3/60. Effective cataract surgical coverage was found to be 43.9% (95% CI: 41.3, 46.5) for those with PVA less than 6/18, 52.3% (95% CI: 49.7, 54.9) for those with PVA less than 6/60, and 55.1% (95% CI: 52.5, 57.7) for those with PVA less than 3/60, as shown in Table 1.

**Table 1:** Cataract Surgical Coverage (persons) and Effective Cataract Surgical Coverage (persons)

Variables	Presenting VA < 3/60	Presenting VA < 6/60	Presenting VA < 6/18
Number of persons with bilateral operable cataract	36	55	116
Number of persons with bilateral pseudo(aphakia), regardless of visual acuity	193	193	193
Number of persons with unilateral pseudo(aphakia) and operable cataract in the other eye.	72	77	108
Cataract Surgical Coverage (%)	88.0	83.1	72.2
No. of persons with unilateral pseudo(aphakia) achieving presenting visual acuity of 6/18 or better, and operable cataract in the other eye	39	43	56
No. of persons with bilateral pseudo(aphakia) achieving presenting visual acuity of 6/18 or better in at least eye one.	127	127	127
Effective Cataract Surgical Coverage (%)	55.1	52.3	43.9

When CSC was calculated across different socio-demographic categories, the highest coverage was observed among individuals aged 50-59 years, followed by those aged 70 years and above. However, there was no statistically significant difference among the age groups in any of the three visual acuity categories. Cataract surgical coverage was found to be higher among females and literate individuals, although these differences were not statistically significant. Among the socio-demographic categories, the APL (Above Poverty Line) group had greater coverage than the BPL (Below Poverty Line) group, except in the PVA category with visual acuity less than 6/60. Among those with PVA less than 6/18, coverage was higher among unmarried or widowed individuals. However, in the other two categories (PVA < 3/60 and PVA < 6/60), coverage was higher among married adults. None of the socio-demographic variables were found to be significantly associated with CSC, as shown in Table 2.

**Table 2:** Cataract Surgical Coverage (persons) with respect to socio-demographic characteristics CSC: Cataract Surgical Coverage, VA: visual acuity.

Variables	n	Presenting VA < 3/60		Presenting VA < 6/60		Presenting VA < 6/18	
		CSC % (95% CI)	p-value	CSC % (95% CI)	p-value	CSC % (95% CI)	p-value
Age (years)							
50–59	36	100.0 (100.0–100.0)	0.855	86.7 (74.5–98.9)	0.966	80.6 (67.7–93.5)	0.764
60–69	164	84.3 (77.7–91.0)		81.0 (74.0–88.0)		67.7 (60.5–74.9)	
≥70	217	88.8 (83.9–93.6)		83.9 (78.4–89.4)		74.2 (68.4–80.0)	
Sex							
Male	163	86.2 (79.7–92.7)	0.854	80.8 (73.8–87.8)	0.801	67.5 (60.3–74.7)	0.488
Female	254	89.1 (84.7–93.5)		84.4 (79.4–89.4)		75.2 (69.9–80.5)	
Marital status							
Married	220	89.1 (84.2–94.0)	0.881	84.9 (79.5–90.3)	0.781	70.9 (64.9-76.9)	0.805
Unmarried/widower	197	86.9 (81.4–92.4)		81.1 (75.0–87.2)		73.6 (67.4–79.8)	
Education							
Illiterate	284	87.3 (82.7–91.8)	0.994	81.1 (75.9–86.2)	0.968	71.8 (66.6–77.1)	0.925
Literate	133	89.7 (83.6 -95.7)		87.4 (81.0–93.8)		72.9 (65.4–80.5)	
Poverty status							
Above poverty line	334	88.1 (84.0–92.2)	0.954	83.0 (78.5–87.5)	0.970	73.4 (68.7–78.1)	0.664
Below poverty line	83	87.7 (79.2–96.2)		83.6 (74.3–92.9)		67.5 (57.4–77.6)	

eCSC was found to be higher among females, married individuals, literate individuals, and those belonging to the APL category compared to their respective counterparts. However, in different age groups, the higher eCSC was found among those aged 70 years or older, except in the VA <6/18 group, where the eCSC was higher among those aged between 50 and 59 years. Like CSC, none of the sociodemographic factors were found to be significantly associated with eCSC, as shown in Table 3.

**Table 3:** Effective Cataract Surgical Coverage (persons) with respect to socio-demographic characteristics eCSC: Effective Cataract Surgical Coverage, VA: visual acuity.

Variables	n	Presenting VA < 3/60		Presenting VA < 6/60		Presenting VA < 6/18	
		eCSC % (95% CI)	p-value	eCSC % (95% CI)	p-value	eCSC % (95% CI)	p-value
Age (years)							
50–59	17	50.0 (30.8–69.2)	0.864	43.3 (25.6–61.1)	0.897	47.2 (30.9–63.5)	0.609
60–69	68	52.2 (43.0–61.3)		50.4 (41.5–59.3)		40.9 (33.3–48.4)	
≥70 years	98	56.9 (49.2–64.5)		54.0 (46.6–61.4)		45.2 (38.5–51.8)	
Sex							
Male	62	49.5 (40.2–58.9)	0.810	47.5 (38.6–56.4)	0.952	38.0 (30.6–45.5)	0.996
Female	121	58.3 (51.4–65.3)		55.1 (48.3–61.9)		47.6 (41.5–53.8)	
Marital status							
Married	101	59.0 (51.3–66.7)	0.828	56.6 (49.1–64.2)	0.810	45.9 (39.3–52.5)	0.793
Unmarried/widower	82	51.0 (42.9–59.2)		47.8 (40.0–55.6)		41.6 (34.7–48.5)	
Education							
Illiterate	114	51.0 (44.1–57.8)	0.927	47.3 (40.7–53.9)	0.921	40.1 (34.4–45.8)	0.875
Literate	69	63.9 (54.4–75.5)		51.4 (53.8–72.4)		51.9 (43.4–60.4)	
Poverty status							
Above poverty line	154	57.8 (51.6–64.0)	0.685	54.5 (48.5–60.6)	0.643	46.1 (40.8–51.5)	0.548
Below poverty line	29	43.9 (31.0–56.7)		42.6 (30.2–55.0)		34.9 (24.7–45.2)	
Total	183	55.1 (49.5–60.8)		52.3 (46.9–57.7)		43.9 (39.1–48.6)	

## Discussion

This was a community-based cross-sectional study conducted in the rural areas of northern India. The CSC was found to be 88.0%, 83.1%, and 72.2%, and the eCSC was found to be 55.1%, 52.3%, and 43.9% in the PVA category, corresponding to less than 3/60, 6/60, and 6/18, respectively.

A national blindness and impairment survey conducted across 31 districts among people aged 50 years and older from 2015 to 2019 found that the CSC was 93.2% among those with a VA of less than 3/60, 89.0% among those with a VA of less than 6/60, and 72% among those with a VA of less than 6/18 [3]. This prevalence was found to be more than in the current study. This might be due to better coverage of cataract surgical services in many districts. This may also be due to the improvement of services over the years. Studies by Limburg et al., Sobti et al., and Patil et al. found the CSC to be less than the current study [6,10,11]. This may be because these studies were conducted before the current study, which represents a period of limited access to cataract surgical services in the past. The CSC was found to be 40.2% in the survey by Sobti et al., which is much lower than the current study, as the age group of participants included is younger (≥40 years) compared to the current research (≥50 years). A study by Bettadapura et al. in Kolar in 2011 demonstrated cataract surgical coverage similar to that in our current study [12].

In this study, none of the socio-demographic factors were found to be significantly associated with CSC. The survey conducted by Bharat et al. found that CSC was related to the age of the participants [13]. Those with higher ages had a higher chance of surgery than those with lower ages. This may be because our study area was relatively more urbanized, with a majority of participants (almost two-thirds) being literate and presumably more aware than in the study by Bharat et al., where the majority of participants were illiterate [13]. Additionally, the differences may be due to variations in the occupations of the participants. Those who were involved in fine works, such as reading and writing, were more likely to visit the ophthalmologist frequently and, therefore, were more likely to be diagnosed with cataracts and undergo early surgery. Thus, the effect of age is expected to vanish in literate adults.

The study by Ramke et al. reported a prevalence of the eCSC ranging from 27.0% to 75.7% [14]. Among all the studied countries, Bangladesh was the only Southeast Asian country and was found to have an eCSC of 35.1%. This was less than the reported eCSC of the current study. This difference might be due to the chronology of surveys conducted and the improvement of services over time. In another study conducted in a district adjacent to our study district, Gurugram in Haryana, which has a predominance of urban areas, higher effective cataract surgical coverage was reported [15].

In our study, we found eCSC to be 43.9%, which is less than the CSC. It would be imperative to investigate poor performance in terms of eCSC. Multiple reasons could contribute to lower eCSC, including sub-optimal quality of cataract surgical services and post-operative care. This stark difference between the two indicators highlights the poor penetration of quality cataract surgical coverage in areas with high CSC [16]. Other possible reasons might be the presence of secondary causes of visual impairment that persisted after the cataract surgery. Similar to our findings on CSC, none of the socio-demographic factors were found to be related to the eCSC.

Our study has some limitations. This was a rapid cross-sectional assessment conducted in rural areas of the Jhajjar district in Haryana. Thus, our findings may not be generalizable to urban areas. The optometrists performed a torchlight and visual examination in an undilated pupil. We did not ascertain and report the cause of poor vision post-surgically among cataract-operated cases. An in-depth exploration will be necessary in these cases to improve the quality of vision outcomes post-surgically. Since eCSC is a vital indicator signifying progress toward achieving universal healthcare and sustainable development goals, investment in quality improvement initiatives for cataract surgical services would be warranted in our study setting.

## Conclusions

The eCSC was found to be 43.9%, which was far lower than the CSC (72.2%). Despite the presence and access to cataract surgical services, lower effective coverage suggests that the quality of cataract surgical services in rural areas of the Jhajjar district in the state of Haryana is suboptimal. Strengthening surgical quality and improving postoperative follow-up care will be imperative for enhancing visual outcomes and effective cataract surgical coverage in this rural population. 
